# Aesthetic Surgical Treatment of Large *Xanthelasma palpebrarum*

**DOI:** 10.1007/s00266-024-04435-x

**Published:** 2024-10-14

**Authors:** Yuan Lin, Liangliang Wu

**Affiliations:** Department of Plastic and Reconstructive Surgery, The First Affiliated Hospital of Wannan Medical College, Wuhu, Anhui Province People’s Republic of China

**Keywords:** *Xanthelasma palpebrarum*, Aesthetic surgical treatment, Eyelid

## Abstract

**Background:**

*Xanthelasma palpebrarum* (XP) is a common eyelid condition. Various treatment modalities exist, each with its own merits and drawbacks. Managing larger lesions poses increased challenges. This study aims to explore the aesthetic surgical management of large *xanthelasma palpebrarum*.

**Methods:**

Patients with *xanthelasma palpebrarum* presenting to our department underwent partial excision of the lesion and debridement of the medullary nucleus under local anesthesia for lesions with a diameter of 6 mm or more. Microsurgical scissors were utilized to remove residual subcutaneous lesions. Postoperatively, tie-over bolster dressings were applied. Patients with lipid and glucose abnormalities received corresponding therapy. Bolster dressings were removed on the third postoperative day, with suture removal seven days later.

**Results:**

All flaps subjected to subcutaneous medullary debridement exhibited complete viability, with minimal localized eyelid depression early postoperatively and inconspicuous scarring three months post-surgery. No instances of lid ectropion were observed. The eyelid maintained a natural appearance, with a low recurrence rate of *xanthelasma palpebrarum*.

**Conclusion:**

Surgical intervention involving partial excision of the lesion and medullary nucleus debridement offers a satisfactory approach for managing large *xanthelasma palpebrarum*.

**Level of Evidence IV:**

This journal requires that authors assign a level of evidence to each article. For a full description of these Evidence-Based Medicine ratings, please refer to Table of Contents or the online Instructions to Authors www.springer.com/00266.

## Introduction

*Xanthelasma palpebrarum* (XP) is the most prevalent form of xanthoma, predominantly manifesting on the eyelids. These lesions typically present as plaque-like, yellow formations, most frequently occurring near the inner canthus. Xanthelasma is considered an idiopathic condition, often showing no significant correlation with hypercholesterolemia [1]. Nonetheless, an increased risk of atherosclerosis can be inferred in hyperlipidemic patients with xanthelasma, attributed to associated abnormalities in lipoprotein or apolipoprotein levels. Various studies have attempted to differentiate between normolipidemic and hyperlipidemic patients exhibiting xanthelasma. Despite these efforts, xanthelasma may not develop in many individuals with hypercholesterolemia, and similarly, these lesions can occur in normolipidemic subjects. Consequently, it has been suggested that xanthelasma may not necessarily be linked to hyperlipidemic conditions. Histologically, XP is characterized by the accumulation of lipid-laden histiocytes within the superficial dermis, predominantly surrounding blood vessels and adnexal structures [2]. While generally benign, XPs can lead to significant cosmetic and functional disfigurement. The medical literature reports various treatment modalities, including laser ablation, TCA (trichloroacetic acid) ablation, and surgical resection. In this context, we introduce a novel approach for the treatment of large XPs, employing a technique of partial lesion removal combined with un-capping microsurgery and inverse peeling.

## Patients

From July 2019 to July 2024, our department treated forty patients diagnosed with *xanthelasma palpebrarum*. The cohort comprised 7 males and 33 females, ranging in age from 26 to 58 years. Among these patients, 10 had dyslipidemia, 3 were diagnosed with diabetes mellitus; while, the remaining individuals exhibited normal lipid profiles.

## Methods

### Preoperative Preparation and Planning

**Fig. 1:**
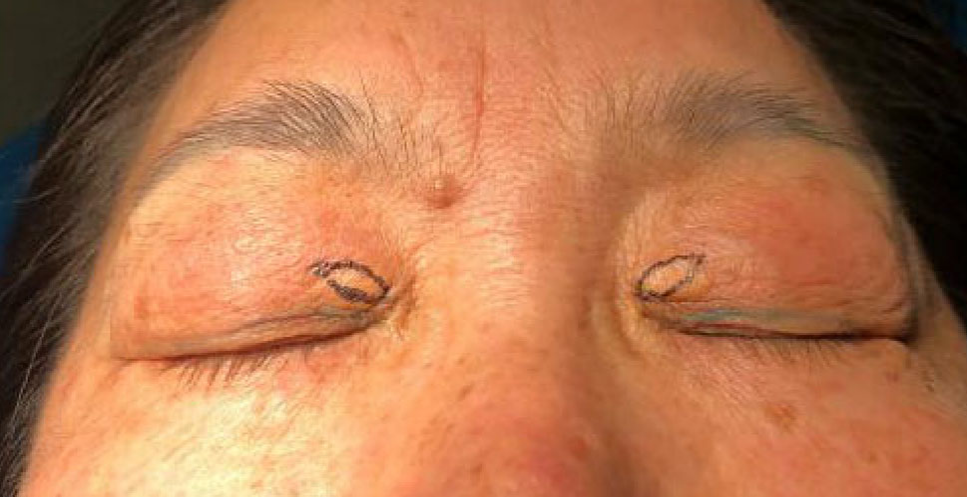
Prior to surgery, an elliptical excision was meticulously planned for the upper segment of the lesion

With the patient in the supine position, the surgeon positioned themselves at the head of the operating table. A crescent-shaped or elliptical excision was delineated around the *xanthelasma palpebrarum* (XP) lesion (Fig. 1). We implemented a modified incision technique, initiating with an upper incision along the margin of the XP and a lower incision line positioned within the lesion. The excised skin’s vertical width ranged from 4 to 6 mm, adjusted based on skin laxity.

### Surgical Procedure

**Fig. 2:**
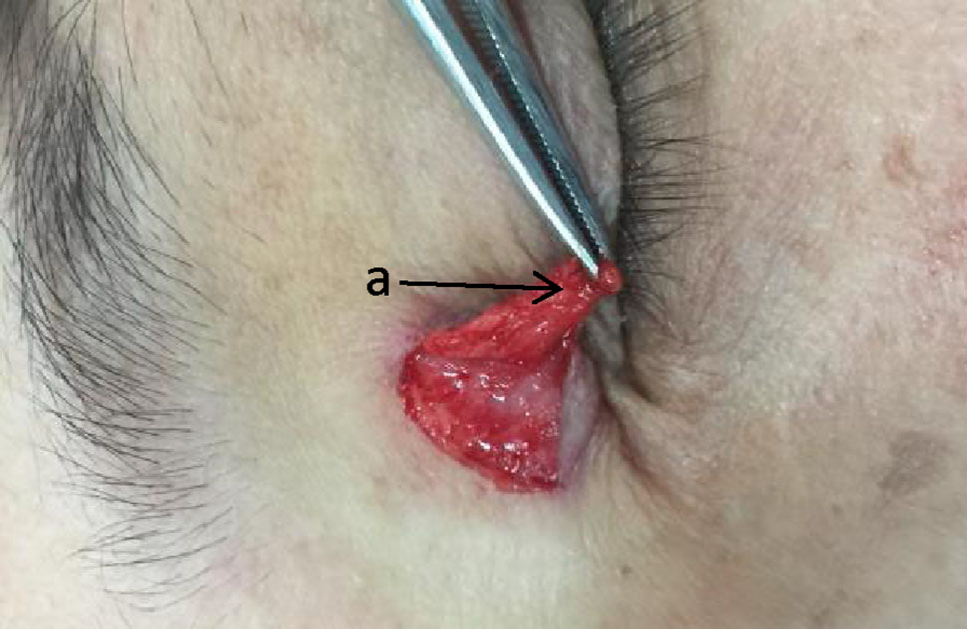
During the un-capping surgery, it was observed that the foam cell tissue (**a**) was located beneath the epidermis

**Fig. 3:**
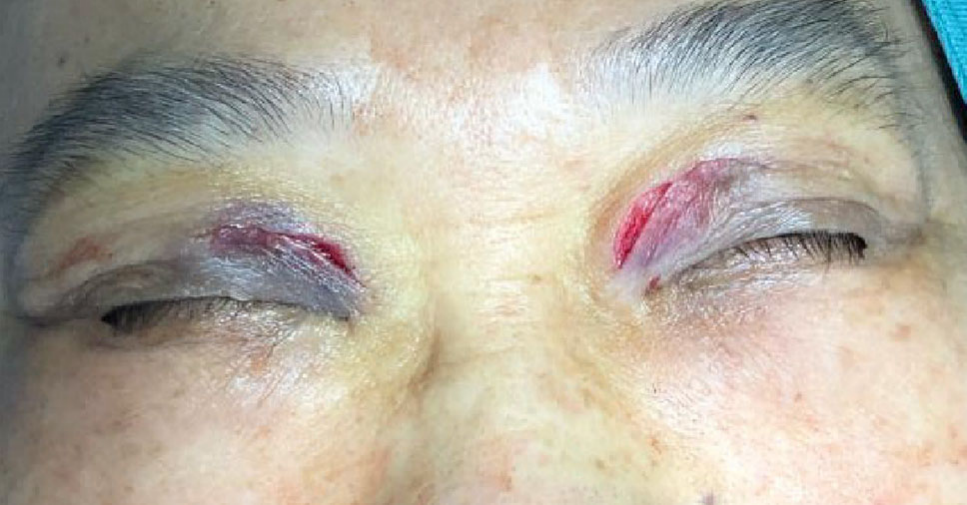
Foam cell tissue in the lower portion was meticulously excised using microscissors subsequent to the removal of the upper segment of the lesion

**Fig. 4:**
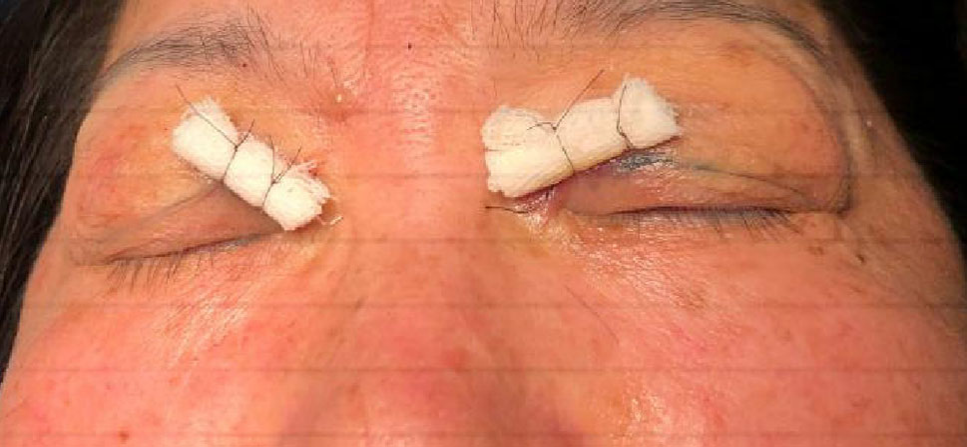
Pressure was meticulously applied using packed Vaseline gauze within the surgical site

Local anesthesia consisting of 1% xylocaine with epinephrine (1:100,000) was sublesionally administered using a 30-gauge needle. After approximately 5 min for the anesthesia to take effect, a scalpel (number 11 blade) was used to incise the skin directly down to the orbicularis oculi muscle, following the upper border of the lesion, then proceeding with the lower incision. The skin pad above the orbicularis oculi was excised, presenting an un-capping procedure (Fig. 2).Subsequently, the lesion was dissected off through the lower incision, underlying the epidermis to the margin of the lesion. The xanthelasma nucleus pulposus was excised from the surface of the orbicularis oculi muscle. Under surgical magnification, foam cells adherent to the flap were meticulously removed with microscissors until normal skin tissue was exposed. Any lipomatous infiltration of the orbicularis muscle was also excised until normal muscle architecture was restored. Due to the removal of cholesterol deposits in the lower aspect of the XP during un-capping, closure of the incision was achieved without tension (Fig. 3).Closure of the incision was performed using 7/0 synthetic nylon sutures. 2 or 3 sutures were placed beneath the orbicularis oculi muscle, and vaseline gauze was applied to secure the skin back to the muscle, resembling a tie-over bolster dressing (Fig. 4).

### Postoperative Precautions and Care

Patients were instructed to maintain cleanliness and dryness of the area postoperatively. Tie-over stitches were removed 2 days after the operation; while, the incision stitches were removed on the 7th postoperative day.

## Results

**Fig. 5:**
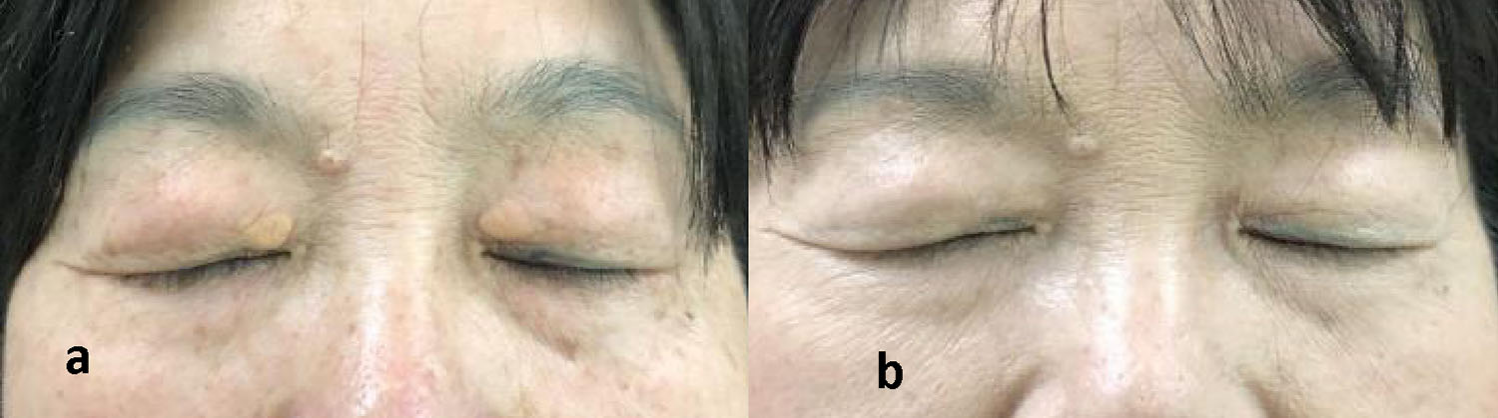
Case1: A patient with large *xanthelasma palpebrarum* near inner canthus. **a** (pre-operative) and **b** (postoperative view one and a half of year later)

**Fig. 6:**
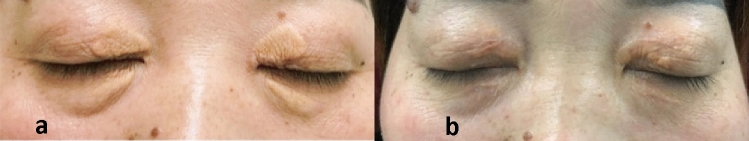
Case2: A patient with large *xanthelasma palpebrarum* on the medial side of the upper and lower eyelids. **a** (pre-operative) and **b** (one year after surgery, there was a slight recurrence below the left upper eyelid)

In all cases, the incisions exhibited robust healing without any occurrence of hematoma beneath the flaps. Patients underwent follow-up examinations for a period ranging from 0.5 to 5 years postoperatively. However, within the first year, recurrence was observed in 5 cases; while, scar formation at the wound site was noted in 6 cases. Remarkably, no complications such as pigmentation abnormalities or ectropion were encountered (Figs. 5, 6, and Table 1).

**Table 1:** Complications and number of patients

Complications	Number of patients	Proportion (%)
Scar	6	15
Hyperpigmentation	0	0
Ectropion	0	0
Relapse	5	12.5
Local depression in the early stage	10	25

## Discussion

*Xanthelasma palpebrarum* stands as the most prevalent form of xanthoma, typically manifesting on the medial aspect of the upper eyelid, with occasional involvement of the adjacent skin near the lower eyelid or the lateral canthus. Characterized by protruding, semi-solid yellow plaques, *xanthelasma palpebrarum* exhibits rare malignant transformations. An array of treatments exists for this condition, encompassing both non-surgical and surgical modalities. Non-surgical interventions comprise cryotherapy, ablation, non-ablation laser therapy, trichloroacetic acid ablation therapy, and Pingyangmycin injection, among others [3–7].

**Fig. 7:**
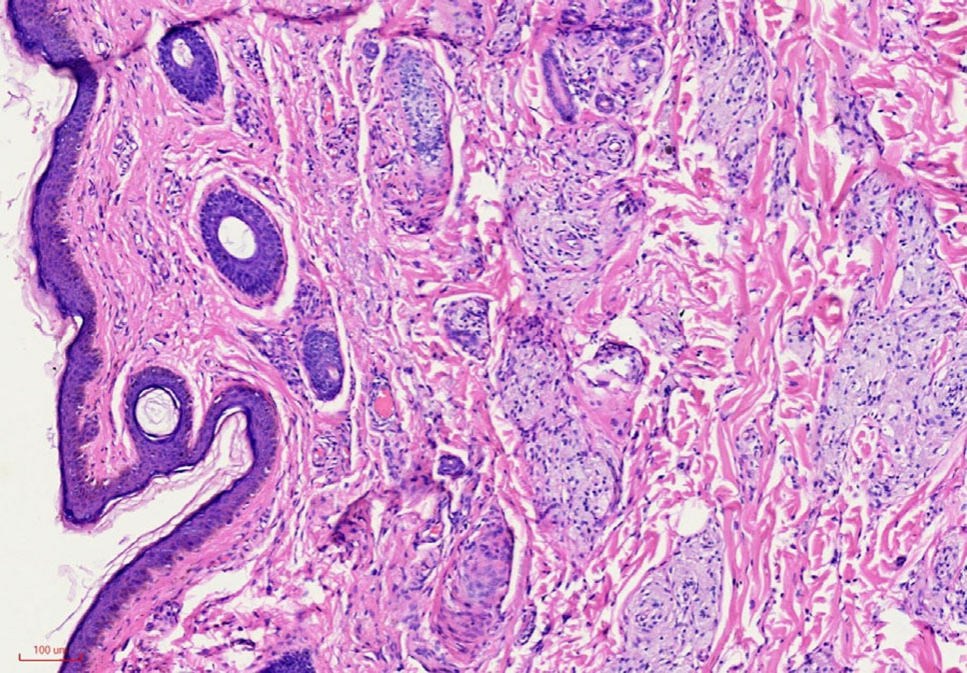
Foam cells were aggregated within the dermis (HE,×100)

Each modality carries its unique set of advantages and drawbacks. Non-surgical approaches, while diverse, may elicit local pigmentation or superficial scarring upon treatment. For smaller lesion areas, various treatment modalities often yield optimal outcomes. However, larger skin lesions pose a challenge, lacking efficacious treatment options devoid of complications. Some researchers have reported favorable outcomes with step-by-step resection, skin grafting, flap transplantation, and similar techniques [8–14]. Additionally, certain scholars advocate for “un-capping” nucleus pulposus removal coupled with flip Nd-YAG laser treatment, citing its simplicity, ease, and low recurrence rates; even in cases of recurrence, this method remains viable for subsequent treatment [14].The pathology of *xanthelasma palpebrarum* is marked by the accumulation of lipid-containing phagocytes, known as foam cells, within the dermis [15] (Fig. 7). While some foam cells may penetrate into the superficial layer of muscle, the epidermis remains intact. This histological characteristic underscores the efficacy of nucleus pulposus removal in eliminating eyelid xanthoma foam cells situated beneath the basal layer of the epidermis. In the “un-capping” procedure, the upper pole of the lesion is incised to the muscle surface to excise the foam cells beneath the skin flap within the lesion area. Subsequently, the flap is inverted, and the nucleus pulposus is meticulously removed using microsurgical scissors, followed by suturing the skin on the lesion surface in situ [16]. Our method represents an advancement in un-capping nucleus pulposus removal. We have devised an elliptical or crescent-shaped excision area for the upper pole of the lesion, enabling the simultaneous removal of the entire skin layer, subcutaneous layer, and superficially infiltrated muscle by foam cells in a single stage. The extent of resection typically ranges from 4 to 6 mm, tailored to the degree of skin laxity. Subsequently, the lower part of lesions that could not be resected in one stage undergoes foam cell removal beneath the epidermis using an operating microscope or magnifying glass to preserve epidermal integrity. This approach effectively minimizes the extent of subcutaneous peeling, thereby reducing operative duration. Due to the epidermis’ lack of inherent blood supply, in situ replantation carries the risk of poor survival. Certain scholars have explored the use of skin grafts obtained during blepharoplasty for treating extensive eyelid xanthoma surgically. Despite local pressure management, six cases reported subcutaneous hematoma or effusion, leading to complications such as skin necrosis [17]. Following nucleus pulposus foam cell removal, we employed 7/0 nylon for incision closure, with additional sutures (2–3 stitches) placed beneath the orbicularis oculi muscle within the dissection range. A Vaseline gauze roll, akin to a tie-over bolster dressing, was then ligated, to be removed after 48 h. This approach effectively mitigates subepidermal effusion or hematoma, fostering skin survival while minimizing inconveniences like eye bandaging. Our surgical method offers several advantages, notably shortened operation times through partial lesion removal in a single stage. Additionally, we effectively prevent subepidermal effusion, thus reducing surgical scars and averting complications such as eyelid ectropion and scar hyperplasia. Therefore, our surgical method can be used as another option besides skin grafting and skin flaps in the surgical treatment of large-area xanthelasma. However, limitations include the potential for residual foam cells beneath the epidermis post-operation, posing a challenge in recurrence control. Moreover, surgery necessitates microscopic or magnifying glass assistance, and entails a recovery period, with early postoperative activities mildly affected.

As of now, the etiology of xanthelasma remains unclear, and a standardized treatment protocol is lacking. The issue of postoperative recurrence persists as a significant challenge, with no definitive method ensuring a zero recurrence rate [11, 18]. Mendelson and Masson reported a recurrence rate of 40% after primary excision of *xanthelasma palpebrarum* (XP), escalating to 60% after secondary excision [19]. In two separate studies, the combination of local flaps and skin grafts derived from blepharoplasty for large-area XP resection yielded recurrence rates of 3% and 8% at 6–12 months post-surgery, respectively [8, 17].

During our follow-up of patients after surgery, 5 patients with recurrence of xanthelasma were found. We observed one recurrence case where the lesion noticeably thickened, causing the epidermis to become extremely thin and difficult to separate. During the process of peeling and trimming the foam cells beneath the epidermis, the epidermis was perforated in efforts to preserve its integrity, potentially leaving behind a small amount of foam cells that contributed to recurrence. Another patient, presenting with large xanthelasma on the upper and lower eyelids, experienced recurrence manifesting as new lesions around the original site, rather than in situ. Additionally, a patient with congenital cleft lip and palate experienced recurrence approximately one year post-surgery, suggesting a potential link to congenital conditions that warrants further investigation. In our study, a 26-year-old patient experienced recurrence six months after surgery despite the absence of dyslipidemia and metabolic diseases, but with a positive family history of xanthelasma. This suggests a familial susceptibility to the condition contributing to recurrence. Another patient, who had hyperglycemia, experienced recurrence around six months post-surgery, indicating a higher likelihood of recurrence among patients with metabolic disorders. Among 40 patients, 10 had abnormal blood lipids before surgery, with an incidence of 25%. The primary issue was elevated LDL, with the highest value being 3.79 mmol/L. Some had elevated TC, with the highest value reaching 7.53 mmol/L; while, others had elevated TG, with the highest value at 2.70 mmol/L. Some patients had only one elevated blood lipid index; while, some patients had multiple elevated blood lipid indexes. Patients who had abnormal blood lipids were treated with oral medications to lower their blood lipids. We did not find a definitive association between abnormal blood lipids and the occurrence of xanthelasma, nor did we find a connection between medical therapy and the rate of postoperative recurrence. Moving forward, we aim to expand our patient cohort, extend follow-up durations, and conduct prospective studies to enhance the rigor and scientific validity of our research.

## References

[1] Parkes ML, Waller TS (1984) *Xanthelasma palpebrarum*. Laryngoscope 94(9):1238–1240 6472020 10.1288/00005537-198409000-00019

[2] Wang KY, Hsu KC, Liu WC et al (2018) Relationship between *Xanthelasma palpebrarum* and Hyperlipidemia. Ann Plast Surg 80:S84–S86 29424765 10.1097/SAP.0000000000001310

[3] Rohrich RJ, Janis JE, Pownell PH (2002) *Xanthelasma palpebrarum*: a review and current management principles. Plast Reconstr Surg 110:1310–1314 12360073 10.1097/01.PRS.0000025626.70065.2B

[4] Heng JK, Chua SH, Goh CL, Cheng S, Tan V, Tan WP (2017) Treatment of *xanthelasma palpebrarum* with a 1064-nm, Q-switched Nd:YAG laser. J American Acad Dermatol 77(4):728–734. 10.1016/j.jaad.2017.03.041 10.1016/j.jaad.2017.03.041 28666611

[5] Sansalone K, Beer K, Pavlis J (2016) Effective treatment of Xanthelasma. J Drugs Dermatol 15(7):891–892 27391641

[6] Goel K, Sardana K, Garg VK (2015) A prospective study comparing ultrapulse CO2 laser and trichloroacetic acid in treatment of *Xanthelasma palpebrarum*. J. Cosm. Dermatol. 14(2):130–139. 10.1111/jocd.12137 10.1111/jocd.12137 25817385

[7] Wang H, Shi Y, Guan H et al (2016) Treatment of *Xanthelasma palpebrarum* with intralesional pingyangmycin. Dermatol Surg 42(3):368–376 26890801 10.1097/DSS.0000000000000660

[8] Lee HY, Jin US, Minn KW, Park Y-O (2013) Outcomes of surgical management of *Xanthelasma Palpebrarum*. Archives Plastic Surg 40(04):380–386. 10.5999/aps.2013.40.4.380 10.5999/aps.2013.40.4.380 PMC3723999 23898435

[9] Khode S, Tan SHT, Tan EA et al (2019) *Xanthelasma palpebrarum*: more than meets the eye. Indian J Otolaryngol Head Neck Surg. 71(Suppl 1):439–446 31742000 10.1007/s12070-018-1345-0 PMC6848656

[10] Choi EJ, Oh TM, Han HH (2020) A modified surgical method combined with blepharoplasty design for treatment of *Xanthelasma palpebrarum*. Biomed Res Int 30(2020):4803168 10.1155/2020/4803168 PMC7721502 33313315

[11] Malekzadeh H, Ormseth B, Janis JE (2023) A practical review of the management of *Xanthelasma palpebrarum*. Plast Reconstr Surg Glob Open 11(5):e4982 37235133 10.1097/GOX.0000000000004982 PMC10208694

[12] Singh A, Tiwary PK, Jha AK et al (2020) Successful treatment of *xanthelasma palpebrarum* with a combination of radiofrequency ablation and wound suturing. J Cosmet Dermatol 19(12):3286–3290 33459474 10.1111/jocd.13678

[13] Yang Y, Sun J, Xiong L, Li Q (2013) Treatment of *xanthelasma palpebrarum* by upper eyelid skin flap incorporating blepharoplasty. Aesthetic Plast Surg 37(5):882–886 23943013 10.1007/s00266-013-0195-0

[14] Levy J-L, Trelles MA (2003) New operative technique for treatment of *xanthelasma palpebrarum*: laser-inverted resurfacing: preliminary report. Ann Plast Surg 50:339–343 12671372 10.1097/01.SAP.0000044249.10349.8D

[15] Bergman R (1994) The pathogenesis and clinical significance of *xanthelasma palpebrarum*. J Am Acad Dermatol 30:236–242 8288783 10.1016/s0190-9622(94)70023-0

[16] Doi H, Ogawa Y (1998) A new operative method for treatment of xanthelasma or xanthoma palpebrarum: microsurgical inverted peeling. Plastic Reconstr Surg 102(4):1171–1174 10.1097/00006534-199809040-00040 9734440

[17] Elabjer BK, Busić M, Sekelj S et al (2009) Operative treatment of large periocular xanthelasma. Orbit 28(1):16–19 19229739 10.1080/01676830802418872

[18] Yee DA, Zhou AE, Khachemoune A (2024) Examining treatment strategies for *xanthelasma palpebrarum*: a comprehensive literature review of contemporary modalities. Arch Dermatol Res 316(5):149 38724802 10.1007/s00403-024-02863-y

[19] Mendelson BC, Masson JK (1976) Xanthelasma: follow-up on results after surgical excision. Plast Reconstr Surg 58(5):535–538 981398

